# UPLC-QTOF/MS-Based Metabolomics Applied for the Quality Evaluation of Four Processed *Panax ginseng* Products

**DOI:** 10.3390/molecules23082062

**Published:** 2018-08-17

**Authors:** Jae Won Lee, Seung-Heon Ji, Bo-Ram Choi, Doo Jin Choi, Yeong-Geun Lee, Hyoung-Geun Kim, Geum-Soog Kim, Kyuil Kim, Youn-Hyung Lee, Nam-In Baek, Dae Young Lee

**Affiliations:** 1 Department of Herbal Crop Research, National Institute of Horticultural and Herbal Science, RDA, Eumseong 27709, Korea; jaewon3@gmail.com (J.W.L.); jiddung3936@gmail.com (S.-H.J.); bmcbr@korea.kr (B.-R.C.); cdj0105@korea.kr (D.J.C.); kimgs0725@korea.kr (G.-S.K.); 2 Department of Oriental Medicine Biotechnology, Kyung Hee University, Yongin 17104, Korea; lyg629@nate.com (Y.-G.L.); zwang05@naver.com (H.-G.K.); nibaek@khu.ac.kr (N.-I.B.); 3 Institute of JinAn Red Ginseng, JinAn 55442, Korea; redgin7@naver.com; 4 Department of Horticultural Biotechnology, Kyung Hee University, Yongin 17104, Korea; younlee@khu.ac.kr

**Keywords:** metabolomics, UPLC-QTOF/MS, *Panax ginseng*, processed products, ginsenosides

## Abstract

In the food industry and herbal markets, it is critical to control the quality of processed *Panax ginseng* products. In this study, ultra-performance liquid chromatography coupled to quadrupole time of flight mass spectrometry (UPLC-QTOF/MS)-based metabolomics was applied for the quality evaluation of white ginseng (WG), tae-geuk ginseng (TG), red ginseng (RG), and black ginseng (BG). Diverse metabolites including ginsenosides were profiled by UPLC-QTOF/MS, and the datasets of WG, TG, RG, and BG were then subjected to multivariate analyses. In principal component analysis (PCA), four processed ginseng products were well-differentiated, and several ginsenosides were identified as major components of each product. S-plot also characterized the metabolic changes between two processed ginseng products, and the major ginsenosides of each product were found as follows: WG (M-Rb1, M-Rb2, M-Rc, Re, Rg1), TG (Rb2, Rc, Rd, Re, Rg1), RG (Rb1, Rb2, Rc, Rd, Re, Rg1), and BG (Rd, Rk1, Rg5, Rg3). Furthermore, the quantitative contents of ginsenosides were evaluated from the four processed ginseng products. Finally, it was indicated that the proposed metabolomics approach was useful for the quality evaluation and control of processed ginseng products.

## 1. Introduction

The root of *Panax ginseng C.A. Meyer*, one of the most critical herbal products in East Asia, has been widely used as ingredients for traditional medicines and functional foods [1,2]. Furthermore, not only raw ginseng but also several processed ginseng products have been used in the food industry and herbal markets [3]. According to the distinct processing methods, there are four processed *P. ginseng* products, white ginseng (WG), tae-geuk ginseng (TG), red ginseng (RG), and black ginseng (BG). Traditionally, WG is produced by dehydrating raw ginseng in sunlight, and TG is manufactured by heating up raw ginseng in 80–95 °C hot water for 20−25 min before drying [4,5]. In the production of RG, raw ginseng is steamed at 95−100 °C for 2−3 h, followed by dehydration [6]. BG is also manufactured by steaming WG for nine cycles at 95−100 °C, for 2−3 h [7,8]. These different methods change the composition of various metabolites in WG, TG, RG, and BG. Ginsenosides are known as the major bioactive components of these ginseng products [9,10]. In particular, the qualitative and quantitative contents of ginsenosides differ depending on the distinct methods used to process raw ginseng [3,11,12]. Dehydration and steaming treatments can induce chemical changes in ginsenosides, which are converted to new bioactive compounds that are not present in raw ginseng [10,13]. For example, WG contains malonyl-ginsenosides (MGR), where a malonyl residue is linked to the glucose of the molecule, while the thermally unstable malonyl residue can be hydrolyzed by a steaming treatment to manufacture RG or BG [14]. Furthermore, BG contains specific ginsenosides, and exhibits more potent pharmacological effects than WG and RG [15]. A variety of ginsenosides have different bioactivities and clinical applications, such as anti-tumor, anti-diabetes, anti-oxidatives, etc. [16,17,18]. Hence, it is critical to assess the levels of ginsenosides for the quality control and effective usage of processed ginseng products.

In the last few decades, more than 150 different ginsenosides have been isolated and identified by nuclear magnetic resonance (NMR) spectroscopy [19,20]. Furthermore, many analytical methods such as high performance liquid chromatography (HPLC) coupled to UV detector [21,22], evaporative light scattering detector (ELSD) [7,23], or mass spectrometry (MS) [24,25] have been used to analyze various ginsenosides from the *P. ginseng* samples. In particular, ultra-performance LC coupled with quadrupole time of flight/MS (UPLC-QTOF/MS) has emerged as a fast and sensitive tool for the comprehensive profiling of ginsenosides [26,27]. A full mass scan mode using QTOF/MS is effective to explore various ginsenosides, as well as many other metabolites in complex mixtures [28]. Recently, for the holistic quality evaluation of processed ginseng products, metabolomics approaches based on UPLC-QTOF/MS have been used to profile various metabolites including ginsenosides [29,30]. Multivariate analyses are used, such as principal component analysis (PCA), which indicates whether any differences exist among samples, and orthogonal partial least squared discrimination analysis (OPLS-DA), which identifies which compounds have been altered for the quality evaluation of commercial WG and RG [30]. A well-constructed metabolomics approach combined with multivariate analyses will be useful for the quality evaluation of other processed ginseng products, such as TG and BG. Until now, many researchers have reported the analysis of WG and RG, whereas there are few studies on the metabolic components of TG and BG.

In this study, we applied the metabolomics approach based on UPLC-QTOF/MS to assess the metabolic compositions of WG, TG, RG, and BG. In the multivariate analyses, several ginsenosides were represented as major components to differentiate these four processed ginseng products. Furthermore, the qualitative and quantitative contents of ginsenosides were characterized for the quality evaluation of WG, TG, RG, and BG.

## 2. Results

### 2.1. Metabolite Profiling of Processed Ginseng Products by UPLC-QTOF/MS

**Figure 1:**
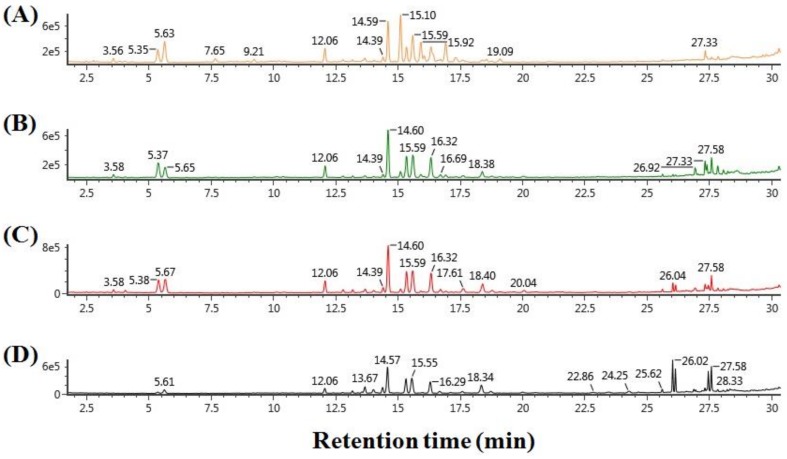
The base peak intensity (BPI) chromatogram of diverse metabolites from (**A**) white ginseng, (**B**) tae-geuk ginseng, (**C**) red ginseng, and (**D**) black ginseng.

UPLC-QTOF/MS with an in-house library, constructed in our previous study [31], was used to profile various metabolites, including ginsenosides, from four kinds of processed ginseng products (i.e., WG, TG, RG, and BG). 70% (*v*/*v*) methanol was used to extract ginsenosides and other metabolites from the four samples. Then, the extracts were subjected to UPLC-QTOF/MS in the negative ion mode. Figure 1 shows the representative base peak intensity (BPI) chromatograms of diverse metabolites from WG, TG, RG, and BG. UPLC separated various metabolites effectively in 30 min. The intensities of several peaks were different depending on the distinct samples. For example, compared to other products, BG showed a lower intensity of peaks eluted from 3 to 6 min, and a higher intensity of peaks eluted from 26 to 28 min in the BPI chromatogram. From 15 to 18 min, WG showed more peaks than other products. These demonstrated that different processing methods changed the metabolic composition of the distinct ginseng products.

### 2.2. Multivariate Analysis to Differentiate Four Processed Ginseng Products

**Figure 2:**
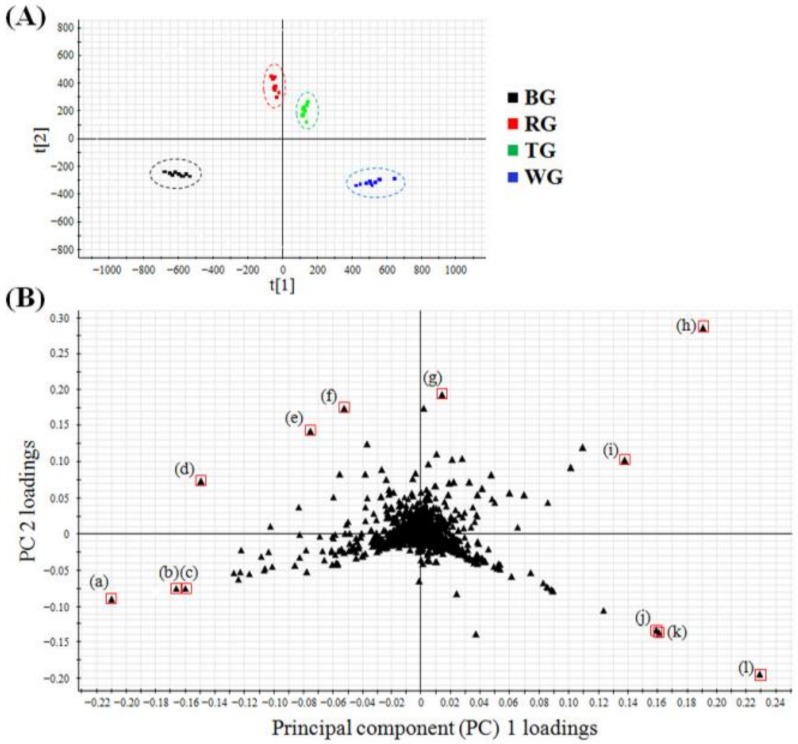
(**A**) The principal component analysis (PCA) score plot and (**B**) loading plot of metabolic datasets of the four processed ginseng products including white ginseng (WG) (*n* = 10), tae-geuk ginseng (TG) (*n* = 10), red ginseng (RG) (*n* = 10), and black ginseng (BG) (*n* = 10). In the PCA loading plot, each point was identified as follows: ginsenoside (a) Rg3, (b) Rg5, (c) Rk1, (d) Rd, (e) Rc, (f) Rb2, (g) Rb1, (h) Rg1, (i) Re, (j) M-Rc, (k) M-Rb2, and (l) M-Rb1. R2X[1] = 0.9443 and R2X[2] = 0.02803.

Next, multivariate data analyses were performed to characterize in detail the distinct composition of various metabolites from WG, TG, RG, and BG. In the metabolite profiling of these four products, the numbers of individual samples were as follows: WG (*n* = 10), TG (*n* = 10), RG (*n* = 10), and BG (*n* = 10). After the UPLC-QTOF/MS-based profiling of individual samples, each dataset was processed using UNIFI software. All of the datasets were then subjected to PCA, to visualize the clustering trends among the four products. In the score plot, four groups of WG, TG, RG, and BG were well-differentiated (Figure 2A). Moreover, in the PCA loading plot, we identified the main variables that functioned to differentiate the four ginseng products in the score plot. Each point represented the *m*/*z*-retention time (RT) pairs of molecules. Based on an in-house library, 14 variables of the loading plot were identified, as well as several ginsenosides (Figure 2B).

**Figure 3:**
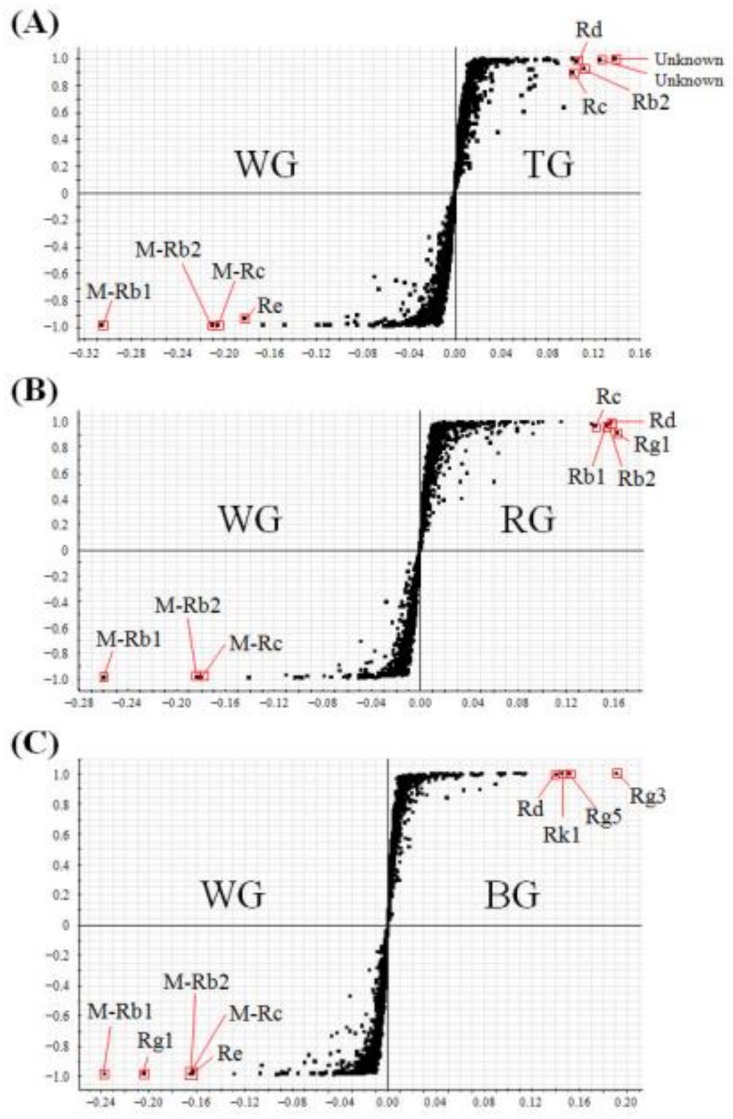
The S-plot of (**A**) white ginseng (WG) vs. tae-geuk ginseng (TG) (*R*^2^: 96%, Q^2^: 92%), (**B**) WG vs. red ginseng (RG) (*R*^2^: 95%, Q^2^: 92%), and (**C**) WG vs. black ginseng (BG) (*R*^2^: 94%, Q^2^: 91%).

OPLS-DA was also performed to discriminate between two selected samples as follows: WG vs. TG, WG vs. RG, WG vs. BG, TG vs. RG, TG vs. BG, and RG vs. BG. An S-plot analysis was then performed to select the critical variables that contributed to distinguish them. In the S-plot, each point showed *m*/*z*-RT pairs of molecules. First, OPLS-DA was performed to compare WG and the other three samples, respectively. In the comparison between WG and TG, the components elevated in WG were showed in the lower left quadrant of S-plot, while the upper right quadrant showed the components elevated in TG (Figure 3A). As a result, four ginsenosides (malonyl (M)-Rb1, M-Rb2, M-Rc, Re) were elevated in WG, while three ginsenosides (Rb2, Rc, Rd) and two unknown compounds were elevated in TG. In the S-plot of WG and RG, it was observed that three MGRs (M-Rb1, M-Rb2, M-Rc) were elevated in WG, while five ginsenosides (Rb1, Rb2, Rc, Rd, Rg1) were elevated in RG (Figure 3B). In the S-plot of WG and BG, five ginsenosides (M-Rb1, M-Rb2, M-Rc, Re, Rg1) were elevated in WG, while four ginsenosides (Rd, Rk1, Rg5, Rg3) were elevated in BG (Figure 3C). In comparison to other three products, WG showed three MGRs (M-Rb1, M-Rb2, M-Rc) as its major components. 

**Figure 4:**
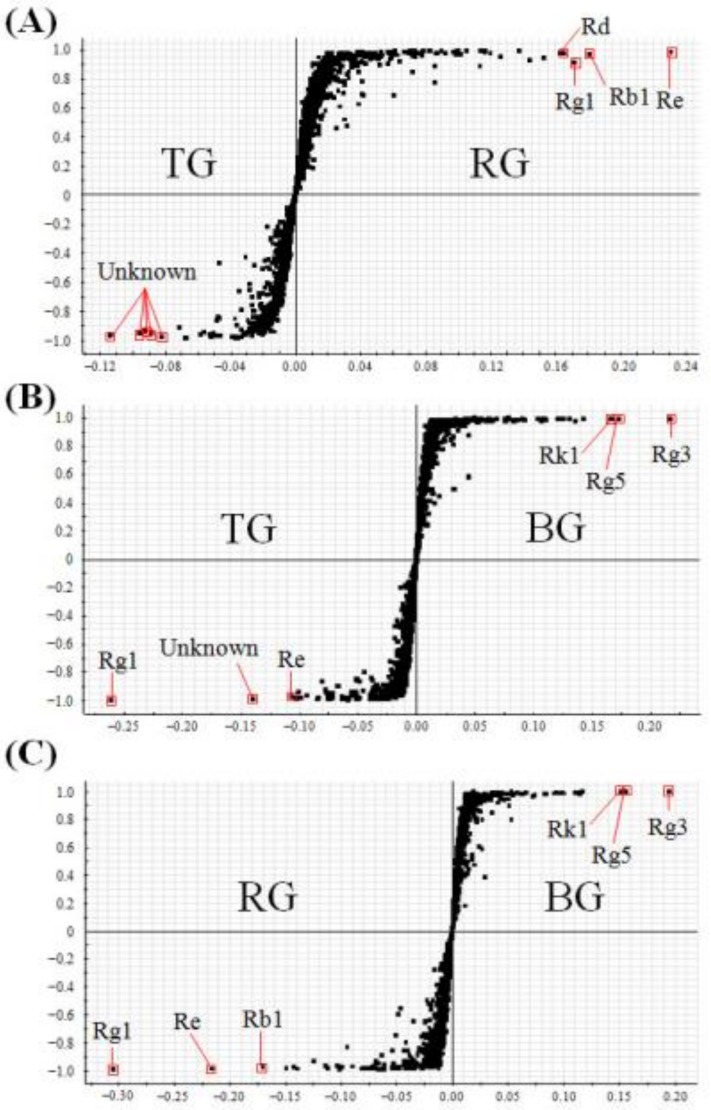
The S-plot of (**A**) tae-geuk ginseng (TG) vs. red ginseng (RG) (*R*^2^: 92%, Q^2^: 89%), (**B**) TG vs. black ginseng (BG) (*R*^2^: 96%, Q^2^: 95%), and (**C**) RG vs. BG (*R*^2^: 94%, Q^2^: 89%).

Second, OPLS-DA was performed to differentiate TG vs. RG, TG vs. BG, and RG vs. BG, respectively. In the S-plot of TG and RG, five unknown compounds were elevated in TG, while four ginsenosides (Rd, Rg1, Rb1, Re) were elevated in RG (Figure 4A). In the S-plot of TG and BG, two ginsenosides (Rg1, Re) and one unknown compound were elevated in TG, while three ginsenosides (Rk1, Rg5, Rg3) were elevated in BG (Figure 4B). In the S-plot of RG and BG, three ginsenosides (Rg1, Re, Rb1) were elevated in RG, while three ginsenosides (Rk1, Rg5, Rg3) were elevated in BG (Figure 4C).

### 2.3. Quantification of Various Ginsenosides from Four Processed ginseng Products

As several ginsenosides were found to be key components for differentiating WG, TG, RG, and BG, it is essential to assess the levels of ginsenosides from each sample. UPLC-QTOF/MS with an in-house library was used to profile ginsenosides from 10 WGs, 10 TGs, 10 RGs, and 10 BGs. UNIFI software was then used to process the datasets of 40 individual samples. Using an in-house library, the numbers of identified ginsenosides from the four products were identified as follows: WG (*n* = 26), TG (*n* = 28), RG (*n* = 34), and BG (*n* = 36). On the basis of a standard curve, the quantification of ginsenosides was also performed in the individual samples. The quantification was based on 10 analyzed biological replicates for each product (*n* = 10), and the values of the amounts were described as mean ± SD (Table 1).

## 3. Discussion

As the processed ginseng products such as WG, TG, RG, and BG have been widely used in the herbal markets, it is critical to perform quality control on these products. For this, in this study, we applied the metabolomics approach to profile various metabolites and to statistically analyze the metabolic datasets for characterizing the four processed ginseng products. First, the previously constructed method based on UPLC-QTOF/MS with an in-house library was used to profile various metabolites including ginsenosides. UPLC provided a fast and high-resolution separation of compounds, and QTOF/MS was effective for detecting various metabolites including ginsenosides, due to its function in the mass scan of whole ions.

Second, multivariate analyses such as PCA and OPLS-DA were effectively used to distinguish and characterize the four processed ginseng products. In a PCA score plot, each point indicates an individual sample. Samples having similar metabolic compositions were scattered close to each other, whereas those having different metabolites were divided. The samples of WG, TG, RG, and BG were well differentiated, and this means that each product has a distinct metabolic composition. WG, TG, RG, and BG were also scattered in order from the right side to the left side. In the processing of *P. ginseng*, the degree of heat treatment to produce the four ginseng products differs (WG < TG < RG < BG). Hence, the metabolic alteration of four ginseng products might be related to the degree of heat treatment. Furthermore, in the PCA loading plot, several ginsenosides were mainly identified as critical variables to differentiate the four ginseng products. This indicated that the ginsenoside contents were mainly affected by the processing methods, including hydration and heat treatment.

Next, in the OPLS-DA and S-plot, the different compositions of metabolites were distinguished between the two selected samples. In the S-plot, the points of the metabolites that greatly contributed to the variance between two groups, were plotted the farther along the x-axis and y-axis. These results showed that several ginsenosides were found to be the main characteristic components of each product. From the data of the S-plot, we identified the characteristic components, which were generally present in the four ginseng products, as follows: WG (M-Rb1, M-Rb2, M-Rc, Re, Rg1), TG (Rb2, Rc, Rd, Re, Rg1, unknown compounds), RG (Rb1, Rb2, Rc, Rd, Re, Rg1), and BG (Rd, Rk1, Rg5, Rg3). Among these processed ginseng products, WG had three MGRs such as M-Rb1, M-Rb2, and M-Rc as its main components. The malonyl residue, which is linked to the glucose in MGRs, is thermally unstable and it easily hydrolyzed by steaming treatment. As WG is manufactured by dehydration in sunlight and with no heat treatment, MGRs are well preserved in WG, compared to other products. Finally, ginsenoside contents were mainly different according to the type of ginseng product analyzed. Hence, it is critical to identify and quantify ginsenosides in order to evaluate the quality of WG, TG, RG, and BG.

By using an in-house library, a number of ginsenosides were identified as follows: WG (*n* = 26), TG (*n* = 28), RG (*n* = 34), and BG (*n* = 36). Furthermore, Table 1 represents the quantitative data of these ginsenosides. Above all, we focused on the ginsenosides that were identified as the main components of each product in the S-plot. For the quantification of three MGRs (M-Rb1, M-Rb2, M-Rc) found as main components of WG, it was necessary to use MGR standards. However, commercially obtaining the MGR standard samples was difficult, due to its thermal weakness and difficulty of separation. Thus, in our laboratory, we isolated these MGRs by ourselves, and quantified them in each sample. In the S-plots of TG, five ginsenosides (Rd, Rc, Rb2, Re, Rg1) were found as its main components. However, RG and BG had higher amounts of these ginsenosides than TG. Hence, they were relative markers for their differentiation from other samples. From the quantification, RG had the highest levels of the five ginsenosides (Rg1, Re, Rb1, Rb2, Rc) found in the S-plots, and of other ginsenosides (gRf, nR2, nR2-R, Rf, Rb3). BG also had the highest levels of the three ginsenosides (Rk1, Rg5, Rg3) found in the S-plots. As these ginsenosides have various pharmaceutical properties, the profiling of ginsenosides should be performed to determine the pharmacological utility of each ginseng product. For instance, TG and RG could display various properties such as anti-cancer (Rc, Rb2), anti-diabetes (Rd, Rb2, Re), and anti-inflammation (Rd, Rb2, Re, Rg1) [17,32,33,34,35]. Furthermore, Rk1, Rg5, and Rg3, which mainly exist in BG, could display the activities of platelet anti-aggregating, anti-inflammation, and anti-cancer [36,37,38,39]. BG had the highest number of ginsenosides among the four ginseng products, and it had five unique ginsenosides (CK, CY, Rh2, Rk2, Rh3). CK, which is known as the metabolite of Rb1, has anti-cancer [40], anti-inflammatory, and hepatoprotective effects [41]. Although other ginsenosides such as Rh2 and Rh3 showed anti-cancer and anti-inflammatory effects [42], there were still no reports on the biological activity of CY and Rk2. In further studies, to characterize in detail the properties of processed ginseng products, quantification of high- or low-abundance ginsenosides is required, as well as isolation and application of specific ginsenosides to find the biological activity.

## 4. Materials and Methods

### 4.1. Standard Constituents and Reagents

HPLC-grade methanol, water, and acetonitrile were purchased from Fisher Scientific Korea (Seoul, Korea). HPLC-grade formic acid was purchased from Fluka Chemie GmbH (Buchs, Switzerland). The ginsenoside standards used in this study were commercially obtained or isolated and purified from *P. ginseng* roots and red ginseng by a series of chromatography procedures, such as silica gel or octadecyl silica gel (ODS) column chromatographies, and the medium-pressure liquid chromatography (MPLC) in our laboratory. The information for 58 ginsenosides has been reported in our previous study [31]. We used these standards for the quality control analysis and the quantification of ginsenosides.

### 4.2. Processed Panax ginseng Samples

**Figure 5:**
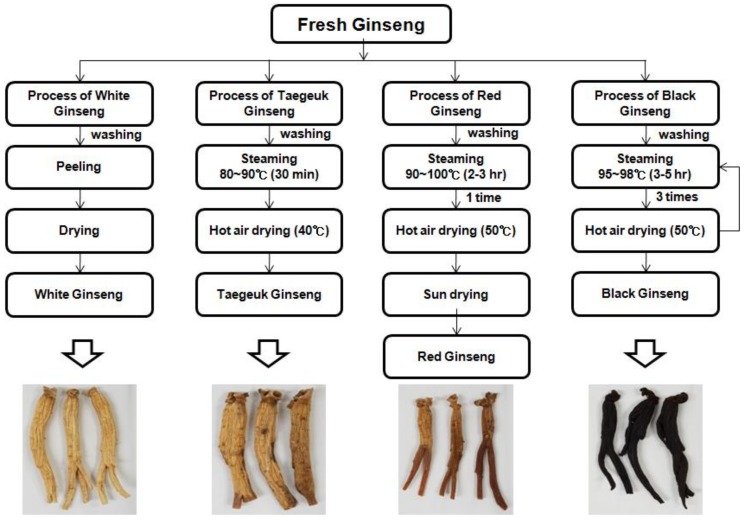
The scheme of producing four processed *P. ginseng* products: white ginseng, tae-geuk ginseng, red ginseng, and black ginseng.

All processed ginseng products were made from five-year-old raw *Panax ginseng* grown in the JinAn, Jeonbuk province (latitude 35°5′ N, longitude 127°45′ E). White ginseng was prepared by washing peeled fresh ginseng and drying it in hot wind or sunlight. Taegeuk ginseng was prepared by first washing the fresh ginseng, and then steaming it for 30 min at 80–90 °C, followed by drying in hot wind and sunlight. The red ginseng was produced by steaming the non-peeled fresh roots of ginseng at 90–95 °C for 3 h and then drying them. Black ginseng was manufactured by triple steaming of the white ginseng at 95–98 °C for 3–5 h in a pottery apparatus, followed by drying at 50 °C for 24 h (Figure 5).

### 4.3. Sample Preparation

Processed white, Taegeuk, red, and black ginseng roots of similar size were selected, and the main and lateral roots were used for experiments after removing the fine roots. The main and lateral roots with average diameters of 2.1 to 2.5 cm, 0.6 to 1.1 cm, respectively, were selected. A mixer (Hanil, Seoul, Korea) was used to grind each sample (<0.5 mm) and the sample was thoroughly mixed; a Retsch MM400 mixer mill (Retsch GmbH, Haan, Germany) was then used to homogenize further subsamples. Fine powder (200 mg) from each sample was suspended in 2 mL of 70% (*v*/*v*) methanol, and ultrasonically extracted for 30 min at 50 °C. After centrifugation (13,500 rpm, 5 min), the solution was filtered by using a syringe filter (0.22 µm) and injected into the UPLC system.

### 4.4. UPLC-QTOF/MS Conditions

For metabolite profiling of the processed ginseng products, we used a previously constructed method based on UPLC-QTOF/MS [31]. UPLC analysis was performed by using a Waters ACQUITY H-Class UPLC (Waters Corp., Milford, MA, USA), and the used column was an ACQUITY BEH C18 column (2.1 mm × 100 mm, 1.7 µm). The temperatures of the column oven and the sample tray were maintained at 40 and 4 °C, respectively. The mobile phase consisted of solvent A (water:formic acid = 100:0.1, *v*/*v*) and solvent B (acetonitrile:formic acid = 100:0.1, *v*/*v*). The gradient elution program was as follows: 0–0.5 min, A 85%; 0.5–1 min, A 85–80%; 1–6 min, A 80%; 6–13 min, A 80–70%; 13–23 min, A 70–65%; 23–24 min, A 65–62%; 24–27 min, A 62–40%; 27–31 min, A 40–10%; 31–32 min, A 10–85%; 32–35 min, A 85%. The flow rate was set at 0.4 mL/min, and the sample injection volume was 2 μL for each run. Next, the TOF MS experiments were performed by using a Waters Xevo G2-S QTOF MS (Waters Corp., Milford, MA, USA). The mass spectrometers performed alternative high- and low-energy scans known as the MS^E^ acquisition mode. The high-accuracy MS data were collected from *m*/*z* 100–2500 Da in negative ion mode. The cone voltage and capillary voltage were 40V and 3.0 kV, respectively. The source and desolvation temperatures were 120 °C and 550 °C, respectively. The cone gas flow and desolvation gas flows were 30 L/h and 800 L/h, respectively. To ensure the mass accuracy and reproducibility of MS condition, a lock spray with leucine-enkephalin (200 pg/mL, 10 μL/min) was used as the reference (*m*/*z* 554.262 (ESI−). For the quality control sample analysis, a standard of Ginsenoside F1 was injected after every analysis of five samples. The precision of LC/MS analysis was determined by a coefficient of variation (CV) for RT (0.1%) and peak intensity (7.9%).

### 4.5. Data Processing and Multivariate Analysis

MS^E^ data were collected and processed using the apex peak detection and alignment algorithms in UNIFI 1.8 (Waters Corp., Milford, MA, USA). The intensity of each ion was normalized by the total ion count to generate a data matrix having RT, *m*/*z* value, and the normalized peak area. Charged species, salt adducts, and fragments were automatically aligned and grouped. The three-dimensional data, including peak number (RT-*m*/*z* pair), normalized peak areas, and sample name, were then exported to EZinfo software 3.0.3 (UMETRICS) for multivariate analyses such as PCA and OPLS-DA. The data were mean-centered and pareto-scaled before PCA and OPLS-DA.

## 5. Conclusions

This study is the first to report the application of an optimal UPLC-QTOF/MS-based metabolomics for the quality evaluation of four processed ginseng products. Multivariate analyses differentiated the metabolic datasets of WG, TG, RG, and BG, and various ginsenosides were found as the characteristic components of each product. As each ginsenoside has specific activities, the ginsenosides profiling can be applied to assess the pharmacological utility of not only ginseng roots, but also processed ginseng products. According to the processing methods that are used to produce WG, TG, RG, and BG, their metabolic composition may change. This information will be useful to optimize the protocol for manufacturing each type of processed ginseng product in the food industry or the herbal medicine market. Finally, this study demonstrated the utility of metabolomics approach for the quality evaluation and control of processed ginseng products. In future, this method will be useful for evaluating processed ginseng products manufactured by using not only the root, but also other parts such as the leaf, stem, and berry.

## Figures and Tables

**Table 1:** Quantification of 39 ginsenosides from four processed *P. ginseng* products such as white ginseng, tae-geuk ginseng, red ginseng, and black ginseng. Values are expressed as mg/g.

No.	RT (min)	Ginsenosides	White Ginseng	Tae-Geuk Ginseng	Red Ginseng	Black Ginseng
1	3.57	20-O-Glucoginsenoside Rf (gRf)	2.2 ± 0.3	1.7 ± 0.2	2.6 ± 0.2	0.17 ± 0.04
2	5.37	Ginsenoside Rg1 (Rg1)	3.9 ± 0.4	4.5 ± 0.3	5.7 ± 0.3	0.63 ± 0.05
3	5.63	Ginsenoside Re (Re)	3.7 ± 0.5	1.7 ± 0.2	4.2 ± 0.4	0.88 ± 0.09
4	11.21	vinaginsenoside R4 (vR4)	0.008 ± 0.004	0.01 ± 0.004	0.024 ± 0.004	0.012 ± 0.004
5	12.05	Ginsenoside Rf (Rf)	1.1 ± 0.2	0.91 ± 0.07	1.4 ± 0.1	0.78 ± 0.04
6	12.78	Notoginsenoside R2 (nR2)	1.3 ± 0.2	1.1 ± 0.1	3.1 ± 0.3	0.9 ± 0.1
7	13.17	Notoginsenoside R4 (nR4)	0.17 ± 0.04	0.18 ± 0.03	0.57 ± 0.06	0.45 ± 0.06
8	13.55	Ginsenoside Rh1 (Rh1)	0.02 ± 0.01	0.09 ± 0.01	0.38 ± 0.03	0.8 ± 0.1
9	13.56	20(R)-Notoginsenoside R2 (nR2-R)	Not detected	Not detected	0.01 ± 0.002	0.004 ± 0.002
10	13.67	Ginsenoside Rg2 (Rg2)	0.16 ± 0.02	0.082 ± 0.006	0.30 ± 0.02	0.66 ± 0.06
11	14.03	20(R)-Ginsenoside Rg2 (Rg2-R)	0.004 ± 0.001	0.01 ± 0.002	0.09 ± 0.01	0.33 ± 0.03
12	14.04	20(R)-Ginsenoside Rh1 (Rh1-R)	Not detected	0.018 ± 0.002	0.11 ± 0.02	0.35 ± 0.04
13	14.39	Ginsenoside Ra2 (Ra2)	3.4 ± 0.2	2.9 ± 0.1	4.9 ± 0.4	5.3 ± 0.4
14	14.56	Ginsenoside Ra3 (Ra3)	1.1 ± 0.2	1.2 ± 0.1	3.1 ± 0.4	4.5 ± 0.5
15	14.59	Ginsenoside Rb1 (Rb1)	4.7 ± 0.6	5.4 ± 0.4	8.9 ± 0.6	4.2 ± 0.4
16	15.1	Malonyl ginsenoside Rb1 (M-Rb1)	6.0 ± 0.7	0.3 ± 0.1	0.32 ± 0.07	Not detected
17	15.35	Ginsenoside Rc (Rc)	1.2 ± 0.2	1.8 ± 0.2	2.9 ± 0.2	2.2 ± 0.3
18	15.55	Ginsenoside Ra1 (Ra1)	1.1 ± 0.3	1.2 ± 0.4	5.5 ± 0.5	7.1 ± 1.5
19	15.6	Ginsenoside Ro (Ro)	0.17 ± 0.02	0.14 ± 0.02	0.19 ± 0.01	0.08 ± 0.01
20	15.91	Malonyl ginsenoside Rc (M-Rc)	1.1 ± 0.2	0.10 ± 0.03	0.04 ± 0.02	Not detected
21	16.31	Ginsenoside Rb2 (Rb2)	0.8 ± 0.1	1.2 ± 0.1	2.0 ± 0.2	1.2 ± 0.1
22	16.7	Ginsenoside Rb3 (Rb3)	0.19 ± 0.05	Not detected	0.45 ± 0.03	0.28 ± 0.09
23	16.92	Malonyl ginsenoside Rb2 (M-Rb2)	1.2 ± 0.2	0.03 ± 0.01	0.014 ± 0.002	Not detected
24	18.39	Ginsenoside Rd (Rd)	0.041 ± 0.005	0.01 ± 0.004	0.5 ± 0.1	0.6 ± 0.1
25	23.43	Ginsenoside Rg4 (Rg4)	Not detected	Not detected	0.11 ± 0.03	0.46 ± 0.05
26	23.54	Compound O (CO)	0.07 ± 0.03	Not detected	0.06 ± 0.03	0.14 ± 0.05
27	23.92	Ginsenoside Rk3 (Rk3)	Not detected	0.04 ± 0.01	0.23 ± 0.04	0.69 ± 0.07
28	24.3	Ginsenoside F4 (F4)	Not detected	0.40 ± 0.03	2.5 ± 0.3	9.0 ± 1.1
29	24.69	Ginsenoside Rh4 (Rh4)	Not detected	0.48 ± 0.07	3.4 ± 0.4	8.9 ± 0.7
30	25.26	Ginsenoside F2 (F2)	0.0020 ± 0.0001	Not detected	0.004 ± 0.002	0.016 ± 0.004
31	26.03	Ginsenoside Rg3 (Rg3)	0.002 ± 0.001	0.044 ± 0.004	0.30 ± 0.02	1.10 ± 0.07
32	26.4	Ginsenoisde Mc (Mc)	Not detected	Not detected	0.01 ± 0.002	0.08 ± 0.02
33	26.56	Compound Y (CY)	Not detected	Not detected	Not detected	0.09 ± 0.02
34	27.38	Compound K (CK)	Not detected	Not detected	Not detected	0.004 ± 0.002
35	27.45	Ginsenoside Rk1 (Rk1)	Not detected	1.60 ± 0.04	4.4 ± 0.3	14.6 ± 1.1
36	27.59	Ginsenoside Rg5 (Rg5)	0.06 ± 0.02	0.41 ± 0.05	2.2 ± 0.2	8.4 ± 0.6
37	27.73	Ginsenoside Rh2 (Rh2)	Not detected	Not detected	Not detected	0.004 ± 0.002
38	29.13	Ginsenoside Rk2 (Rk2)	Not detected	Not detected	Not detected	0.012 ± 0.002
39	29.14	Ginsenoside Rh3 (Rh3)	Not detected	Not detected	Not detected	0.036 ± 0.006

## Abbreviations

UPLC	Ultra-performance liquid chromatography
QTOF	Quadrupole time of flight
ESI	Electrospray ionization
MS	Mass spectrometry
LOD	Limits of detection
RT	Retention time
